# Identification and comparative analysis of subolesin/akirin ortholog from *Ornithodoros turicata* ticks

**DOI:** 10.1186/s13071-015-0749-x

**Published:** 2015-02-28

**Authors:** Hameeda Sultana, Unnati Patel, Daniel E Sonenshine, Girish Neelakanta

**Affiliations:** Center for Molecular Medicine, College of Sciences, Old Dominion University, Norfolk, 23529 VA USA; Department of Biological Sciences, Old Dominion University, Norfolk, 23529 VA USA

**Keywords:** Subolesin, Anti-vector vaccine, Phylogenetic analysis, Relapsing fever, Nucleotide and amino acid sequence alignment, Gene expression, Post-translational modifications

## Abstract

**Background:**

Subolesin is an evolutionary conserved molecule in diverse arthropod species that play an important role in the regulation of genes involved in immune responses, blood digestion, reproduction and development. In this study, we have identified a subolesin ortholog from soft ticks *Ornithodoros turicata*, the vector of the relapsing fever spirochete in the United States.

**Methods:**

Uninfected fed or unfed *O. turicata* ticks were used throughout this study. The subolesin mRNA was amplified by reverse transcription polymerase chain reaction (RT-PCR) and sequenced. Quantitative-real time PCR (QRT-PCR) was performed to evaluate subolesin mRNA levels at different *O. turicata* developmental stages and from salivary glands and gut tissues. Bioinformatics and comparative analysis was performed to predict potential post-translational modifications in *O. turicata* subolesin amino-acid sequences.

**Results:**

Our study reveals that *O. turicata* subolesin gene expression is developmentally regulated, where; adult ticks expressed significantly higher levels in comparison to the larvae or nymphal ticks. Expression of subolesin was evident in both unfed and fed ticks and in the salivary glands and midgut tissues. The expression of subolesin transcripts varied in fed ticks with peak levels at day 14 post-feeding. Phylogenetic analysis revealed that *O. turicata* subolesin showed a high degree of sequence conservation with subolesin’s from other soft and hard ticks. Bioinformatics and comparative analysis predicted that *O. turicata* subolesin carry three Protein kinase C and one Casein kinase II phosphorylation sites. However, no myristoylation or glycosylation sites were evident in the *O. turicata* subolesin sequence.

**Conclusion:**

Our study provides important insights in recognizing subolesin as a conserved potential candidate for the development of a broad-spectrum anti-vector vaccine to control not only ticks but also several other arthropods that transmit diseases to humans and animals.

**Electronic supplementary material:**

The online version of this article (doi:10.1186/s13071-015-0749-x) contains supplementary material, which is available to authorized users.

## Background

Ticks are blood-feeding arthropods that are classified into three families: (a) Ixodidae commonly referred as “hard ticks” (b) Argasidae known as “soft ticks” and (c) Nuttalliellidae [1,2]. A total of 694 species of hard ticks, 177 species of soft ticks and single species of Nuttalliellidae have been confirmed to be present in nature [1-3]. Several of the “hard” and “soft” ticks can transmit various disease causing organisms to humans and animals [3,4]. These two groups of ticks have evolved different blood feeding behaviors [1,3]. Ixodid ticks feed on a vertebrate host for a longer duration of time, while, Argasid ticks feed within an hour [1,3]. Differences in the feeding behaviors show that these ticks have evolved unique mode of blood feeding strategies that must be taken into account when designing vaccines for disrupting transmission of the tick-borne pathogens to humans or animals.

Control strategies for tick infestations basically involve use of acaricides that are shown to be ineffective in many instances [5]. The recent development of recombinant Bm86 protein-based anti-vector vaccines, TickGARD and GAVAC against *Rhipicephalus microplus* ticks showed only limited success due to its variable efficacy against some strains of this tick species [6-9]. Therefore, identification and characterization of new protective antigens that are conserved and effective against various tick strains and species remains important. Recently, Subolesin a tick-protective antigen that is a structural and functional ortholog of insect and vertebrate akirins was identified in *Ixodes scapularis* ticks [10-12]. To date, Subolesin from more than 15 tick species have been identified and characterized [12,13].

Subolesin/akirins family proteins are evolutionary conserved transcriptional factors in arthropods that regulate expression of genes involved in immune responses, blood digestion, reproduction and development [12,14]. RNA interference (RNAi)-mediated silencing of Subolesin gene expression and immunization trials using recombinant Subolesin protein have demonstrated protective efficacy against tick infestations, reduced vectorial capacity and fertility of several arthropod species [12,14-17]. In addition, immunization of White-tailed deer with recombinant Subolesin resulted in significant protection (83%) from tick infestations [18]. Collectively, these studies highlight Subolesin as an important candidate for developing a single anti-vector vaccine against a wide range of arthropods.

Argasid ticks including *Ornithodoros turicata, O. moubata and O. erraticus* are competent vectors for the causative agents of human relapsing fever [19]. *O. turicata* is distributed in the Midwestern and Southwestern United States*, O. erraticus* in the Mediterranean basin and southern Europe and *O. moubata* in South and East Africa and Madagascar [19-21]. In addition to the transmission of human pathogens, *O. turicata* ticks are also suggested to be competent vectors for the transmission of canine jaundice agent *Leptospira pomona* [22]. Recently, subolesin orthologs were identified in *O. moubata and O. erraticus* ticks [13]. Vaccination studies with the subolesin proteins induced a partial protective effect resulting in reduced oviposition rates in these ticks [13]. In this study, we aimed to identify and characterize subolesin ortholog from *O. turicata* that are principal vectors for human relapsing fever borreliae in the United States. This study provides important insights for the growing evidences in the development of subolesin as a broad-spectrum anti-vector vaccine against a wide range of arthropods.

## Methods

### Ticks

Uninfected *O. turicata* ticks were used throughout this study. Larvae, nymphs and adult female ticks used in this study were reared from specimens originally collected from burrows of the gopher tortoise (*Gopherus polyphemus*) in Florida, U.S.A. (donated by Dr. J.H. Oliver, Jr., Georgia Southern University, Statesboro, GA). The colony was maintained continuously at the Department of Biological Sciences, Old Dominion University. All use of animals in this study was carried out in strict accordance with the recommendations in the Guide for the Care and Use of Laboratory Animals of the National Institute of Health. The protocol used in this study (permit number: 10–018) was approved by the Old Dominion University Institutional Animal Care and Use Committee (Animal Welfare Assurance Number: A3172-01). Animal husbandry was provided under the Association for Assessment and Accreditation of Laboratory Animal Care Program at ODU. Acepromazine tranquilizer was administered to the animals prior to handling to minimize anxiety and/or discomfort prior to or during tick feeding. To generate fed ticks, unfed ticks were fed on naïve 6–8 weeks old CD1 mice (Charles River laboratories). Ticks were collected upon repletion and processed for RNA extraction. All ticks were housed in the controlled environment chamber (Paramter Generation and Control, Black Mountain, NC) at 23°C with 95% relative humidity and a 14/10 h light/dark photoperiod regiment.

### Quantitative real-time PCR (QRT-PCR) analysis

Total RNA from larvae, nymphs and adult ticks was generated using the Qiagen RNeasy kit (Qiagen, Valencia, USA) following the manufacturer’s instructions. RNA was converted to cDNA using BioRAD cDNA synthesis kit (BioRAD, Hercules, USA). The generated cDNA was used as a template for quantifying subolesin transcripts using oligonucleotides 5′ GCTGCCAACATTCGGGAAGA 3′ and 5′ GACGAAAGGTGAACAGAGGTTGGT 3′. As an internal control and to normalize the amount of template, *O. turicata* 28S transcripts were quantified using oligonucleotides 5′ GATTCCCACTGTCCCTATCTACTATCT 3′ and 5′ GCGACCTCCCACTTATGCTACA 3′. QRT-PCR was performed using iQ-SYBR Green Supermix (Biorad, USA). Standard curve was prepared using 10-fold serial dilutions starting from 1 ng to 0.00001 ng of known quantities of *subolesin or 28S* fragments and QRT-PCR reactions were performed as described [23,24].

### PCR, cloning and sequencing of *O. Turicata* subolesin

Total RNA generated from freshly unfed or fed *O. turicata* ticks were processed for cDNA synthesis. The obtained cDNA was used as template for the amplification of subolesin gene. Following are the published oligonucleotides [13] used for the PCR amplification of subolesin transcripts (F 5′ ATGGCTTGYGCRACATTAAAGCGRAC 3′ and R 5′ TTTGGTCGTASGTAAAYTTRACAAATGTG 3′). PCR was performed using the following conditions: Initial denaturation at 94 degrees for 3 min followed by 35 cycles of steps including 94 degrees for 40 sec, 58 degrees for 40 s and 72 degrees for 1 min 30 sec. PCR reactions were later run on 1% agarose gels and corresponding band (~470 bp) was purified using Qiagen Gel Extraction Kit (Qiagen, USA). Next, the PCR product was ligated into pGEMT-Easy vector (Promega, USA) and transformed into *E. coli* DH5 alpha competent cells. Transformed cells were plated on LB Agar plates containing Ampicillin (50 micrograms/ml) and clones were selected for plasmid preparation using Qiagen mini prep kit (Qiagen). At least three independent positive clones were sequenced from both ends at the Simple Seq core facility (Eurofins MWG Operon Inc., Huntsville, USA) using standard M13 F and M13 R oligonucleotides. Alignment of nucleotide sequences and annotated amino acid sequences of all three clones are shown in Additional file 1: Figure S1.

### Prediction of subolesin/akirins ortholog(s) post-translational modification and localization

GenBank accession numbers for the sequences used in the study are: *A. aegypti* (XP_001662294), *A. albopictus* (ACF49499), *A. americanum* (ABA62326), *A. gambiae* (XP_308938), *A. hebraeum* (ABY84524), *C. quinquefasciatus* (XP_001863200), *D. marginatus* (ABA62333), *D. variabilis* (AAV67034), *G. morsitans* (ADD20629), *H. longicornis* (ACA84004), *H. marginatum* (ABA62335), *H. punctata* (ABA62336), *H. qinghaiensis* (ACA09713), *I. ricinus* (ABA62325), *I. scapularis* (XP_002414493), *O. erraticus* (ADN66054), *O. moubata* (ADN66053), *O. turicata* (KP708703), *R. appendiculatus* (ABA62331), *R. microplus* (ABZ89745) and *R. sanguineus* (ABA62332). These sequences were downloaded from GenBank and individually analyzed at PROSITE (http://prosite.expasy.org/) for predicting post-translational modifications such as Protein kinase C and Casein kinase II phosphorylation sites, myristoylation, glycosylation and serine rich region sites.

### Salivary gland and gut dissection

Salivary glands and gut tissues were dissected from individual freshly fed adult female ticks in sterile 1× phosphate buffer saline. These tissues were individually homogenized in RLT buffer (Qiagen) and processed for RNA extraction using the Qiagen RNA extraction kit following the manufacturer’s instructions. RNA samples were later converted to cDNA using BioRAD cDNA synthesis kit followed by QRT-PCR using iQ-SYBR Green Supermix (BioRAD, USA).

### Statistics

Statistical significance in the data sets was analyzed using GraphPad Prism4 software and Microsoft Excel. For data to compare two means, the non-paired Student *t* test was performed. P values of <0.05 were considered significant in all tests. Wherever necessary, statistical test and P values used are reported.

## Results

### Amplification, cloning and sequencing of subolesin homolog in *O. Turicata* ticks

**Figure 1:**
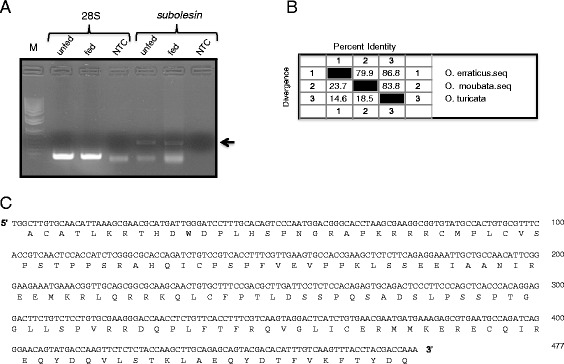
**PCR amplification, nucleotide and deduced amino acid sequence of** ***O. turicata*** **subolesin gene. A)** PCR amplification of subolesin gene is shown. Band of approximately 470 bp was evident in both unfed and fed *O. turicata* ticks. The 28S rRNA levels were shown as controls. Arrow indicates subolesin PCR fragments. **B)** Percent identity (horizontally above black boxed diagonal) and divergence (vertically below black boxed diagonal) of *O. turicata* subolesin nucleotide sequence in comparison to *O. erraticus* and *O. moubata* subolesin sequences is shown. **C)** The nucleotide sequence of *O. turicata* subolesin gene was obtained by sequencing PCR products cloned in the pGEMT-easy vector. The deduced amino acid sequence is shown as a single letter code below the nucleotide sequence.

The Subolesin/akirins are evolutionary conserved transcriptional factors in diverse arthropod species [12]. To identify whether *O. turicata* ticks also encode and express subolesin, cDNA prepared from unfed and fed *O. turicata* ticks was used as the template for PCR amplification. A band of approximately 470 bp was evident in both unfed and fed samples (Figure 1A). The band from the fed tick sample was excised and processed for sequencing as described in the Methods section. Among the three clones that were sequenced, one of the clones had guanine and the other two clones had adenine nucleobase at position 11 (Additional file 1: Figure S1A). However, these differences in the nucleotide sequences did not affect any change in the annotated amino acid sequences (Additional file 1: Figure S1B). No changes were seen in the rest of the nucleotide sequence of all three clones (Additional file 1: Figure S1A). The subolesin nucleotide sequence from clone one (GenBank accession number: KP708703) was considered for further analysis. Analysis of the nucleotide sequence revealed that the *O. turicata* sequence showed more than 83% and 86% identity with *O. moubata* and *O. erraticus* subolesin sequences respectively (Figure 1B). The subolesin nucleotide sequence was annotated based on the predicted coding region of subolesin orthologs from various hard ticks (Figure 1C).

### Comparison of *O. Turicata* subolesin sequence with other known arthropod orthologs

**Figure 2:**
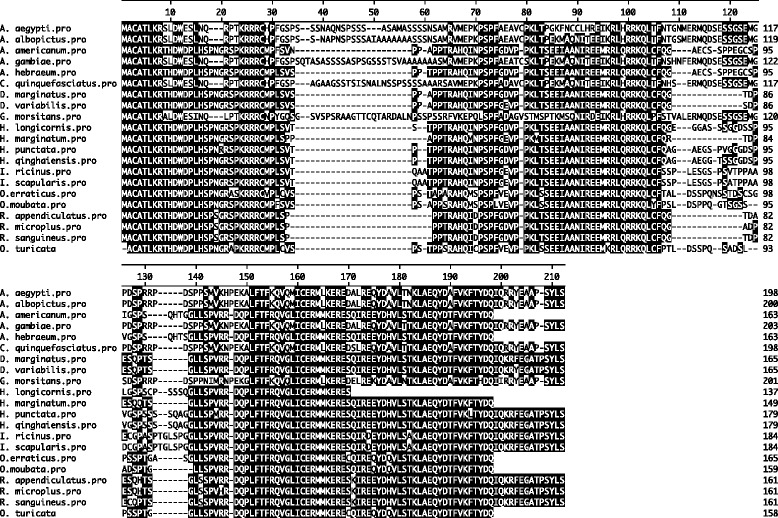
**Alignment of** ***O. turicata*** **subolesin amino acid sequence with other soft ticks, hard ticks and Diptera subolesin sequences.** Amino acid sequence alignment using Clustal W program in DNASTAR Lasergene is shown. Residues that match are shaded in black color. GenBank accession numbers for the sequences used are mentioned in the Methods section.

**Figure 3:**
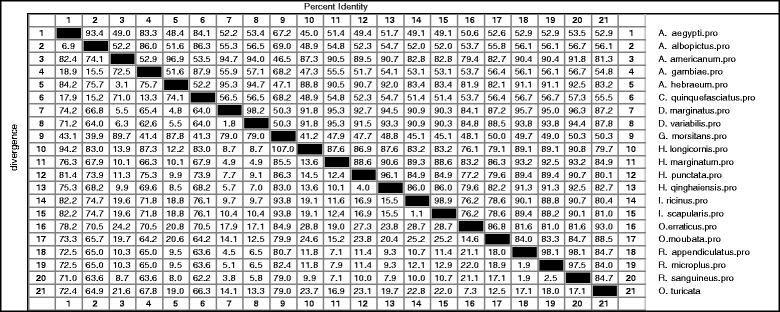
**Amino acids sequence distances of** ***O. turicata*** **subolesin with other soft ticks, hard ticks and Diptera subolesin sequences.** Percent identity (horizontally above black boxed diagonal) and divergence (vertically below black boxed diagonal) of *O. turicata* subolesin in comparison to other subolesin sequences is shown. Sequence distances data was generated based on the Clustal W alignment of the sequences. GenBank accession numbers for the sequences used are mentioned in the Methods section.

**Figure 4:**
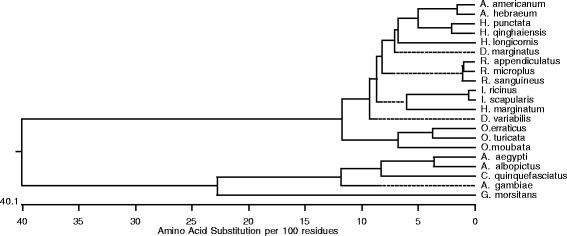
**Phylogenetic analysis of** ***O. turicata*** **subolesin with soft ticks, hard ticks and diptera subolesin amino acid sequences.** Phylogenetic analysis was performed in DNASTAR by ClustalW slow/accurate alignment method using Gonnet as default value for protein weight matrix. Scale shows amino acid substitution per 100 residues. *O. turicata* subolesin sequence comes within the same clade with other soft and hard ticks. GenBank accession number for each sequences are mentioned in the Methods section.

The subolesin/akirin primary amino acid sequences from thirteen hard ticks, two soft ticks, four mosquito species and one from *Glossina morsitans* were downloaded from National center for Biotechnology information (NCBI). Alignment of deduced amino acid sequence of *O. turicata* with other sequences using the Clustal W program revealed a high degree of conservation across the entire amino acid sequences (Figure 2). The *O. turicata* subolesin amino acid sequence shares approximately 50% identity with *G. morsitans*, 50-56% identity with several mosquito species, 79-88% identity with several hard ticks, 88.5% identity with *O. moubata* and 93% identity with *O. erraticus* ticks (Figure 3). The phylogenetic analysis of deduced subolesin amino acid sequences showed that *O. turicata* sequence comes within the same clade as other soft ticks (*O. erraticus* and *O. moubata*) and hard ticks. *Glossina* and mosquito subolesin sequences formed a different clade that was separated from hard and soft ticks (Figure 4).

### Post-translational modification predictions in subolesin/akirins orthologs

**Figure 5:**
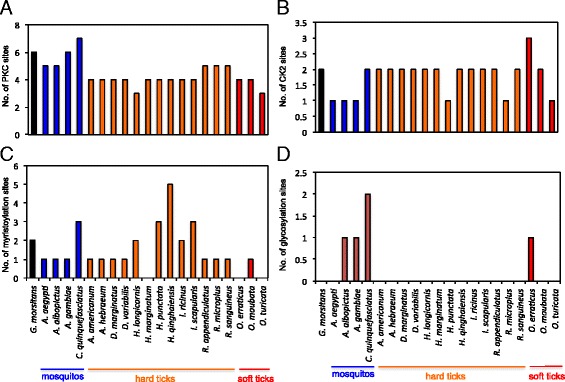
**Prediction and comparative analysis of** ***O. turicata*** **subolesin post-translational modifications.** Annotated amino acid sequences of several subolesins were individually analyzed in PROSITE database for PKC phosphorylation **(A)**, CK2 Phosphorylation **(B)**, Myristoylation **(C)** and Glycosylation **(D)** sites. Histograms represent number of post-translational modification sites for each Subolesin. Organism names and groups are shown at the bottom of the figure.

To predict conserved and/or unique post-translational modifications, subolesin amino acid sequences from hard and soft ticks, mosquitos and *Glossina* species were analyzed by PROSITE as described in the Methods section. Based on the PROSITE analysis, all subolesin sequences carried more than two Protein kinase C (PKC) phosphorylation sites (Figure 5A). *C. quinquefasciatus* subolesin sequence showed higher numbers (seven) and *H. longicornis* and *O. turicata* ticks showed lower numbers (three) of PKC phosphorylation sites (Figure 5A). In addition, all subolesin sequences carried at least one Casein kinase II (CK2) phosphorylation site (Figure 5B). The *O. erraticus* soft ticks carried higher numbers (three) and three mosquito species (*A. aegypti, A. albopictus, A. gambiae*) and *O. turicata* ticks carried one CK2 phosphorylation site (Figure 5B). The number of myristoylation sites in the subolesin sequences varied among different species (Figure 5C). One hard tick (*H. marginatum*) and two soft ticks (*O. erraticus* and *O. turicata*) had no myristoylation sites in the sequence (Figure 5C). Whereas, hard tick *H. qinghaiensis* carried large number of myristoylation sites (five) in comparison to all other arthropod species (Figure 5C). Prediction of glycosylation sites in different subolesins revealed that, except *A. aegypti* all other mosquito subolesins that were analyzed carried at least one glycosylation site in the sequence (Figure 5D). Among hard and soft ticks, *O. erraticus* is the only tick that carried glycosylation site in the subolesin sequence (Figure 5D). Among all the members, the *A. aegypti* and *A. gambiae* subolesin’s are the only two that carry serine-rich domain at N-terminus of the sequence (Additional file 1: Figure S2). Collectively, predictions of the post-translational modifications revealed interesting insights for the molecular function of subolesins/akirins in various arthropod species that transmit human pathogens.

### Expression of *O. Turicata* subolesin is developmentally regulated

**Figure 6:**
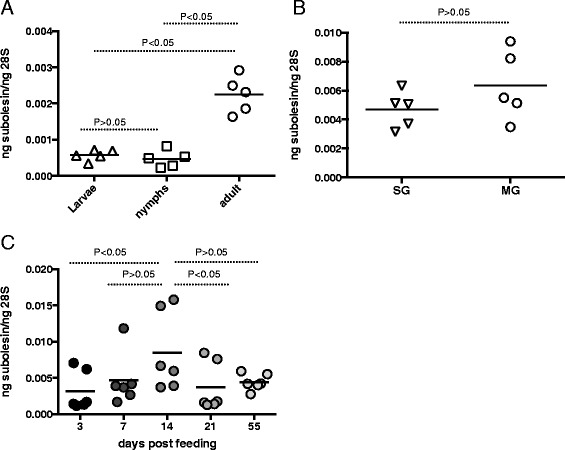
**Expression of** ***O. turicata*** **subolesin is developmentally regulated. A)** Total RNA from unfed larvae, nymphs and female adults was prepared and amount of *O. turicata* subolesin transcripts were quantified by QRT-PCR normalized to *O. turicata* 28S levels. Each triangle/square/circle represents one individual tick. **B)** Expression of subolesin transcripts in salivary glands (SG) and midguts (MG) isolated from fed adult female *O. turicata* ticks are shown. Each inverted triangle/circle represent one individual tissues sample isolated from one tick. **C)** QRT-PCR analysis of subolesin mRNA levels in fed *O. turicata* female ticks at different days post-feeding is shown. Each circle represents one tick. Statistics was performed using the Student’s *t* test and the P value is shown.

We determined whether Subolesin was regulated during *O. turicata* development. The subolesin mRNA levels were assessed by Quantitative real-time PCR using *O. turicata* 28S as an internal control. The subolesin was expressed at lower levels in larvae and nymphs but was significantly (P < 0.05) increased in adults (Figure 6A). To assess whether subolesin gene expression is differentially regulated in different tick tissues upon feeding, salivary gland and gut tissues were separately isolated from fed ticks and processed for RNA extraction and QRT-PCR analysis. We found no differences in the levels of subolesin transcripts between gut and salivary gland tissues that were isolated from fed ticks (Figure 6B). In addition, analysis of gene expression on different days post feeding revealed that in adult ticks the expression of *O. turicata* subolesin mRNA levels peaks up at day 14 post feeding in comparison to the early or late time points after feeding (Figure 6C). Collectively, these results revealed that subolesin expression is developmentally regulated, ubiquitously expressed in both salivary glands and gut tissues and suggest its role in blood digestion in *O. turicata* ticks.

## Discussion

The development of Bm86-based vaccines has raised enormous interest among researchers to identify conserved antigens in ticks that can be considered as a single vaccine candidate to control multiple tick infestations [25-27]. However, it has been noted that the sequence variations in Bm86 orthologs caused variable responses of vaccination between various tick species and strains [6-9]. These studies suggested for the development of a better cross-protective vaccine against wide range of tick species. As subolesin (a conserved antigen) has been shown to be protective against different tick species [12,15,16], in this study, we have identified and characterized its ortholog from *O. turicata* ticks that transmit relapsing fever spirochete in the United States.

The *O. turicata* subolesin ortholog show a high degree of sequence identity with its soft tick counterpart’s *O. erraticus* and *O. moubata* and with the hard ticks at both nucleotide and amino acid sequence levels (Figures 2 and 3). Recent study from de la Fuente et al., used *R. microplus* subolesin in a yeast two-hybrid screen and have identified two subolesin-interacting proteins containing transduction/transcription domain and suggested involvement of subolesin in tick gene transcription [14]. Our phylogenetic analysis reveal that *O. turicata* subolesin groups within the same clade with other two sequenced soft ticks and several hard ticks suggesting its role in gene transcription in a similar manner as reported for the hard ticks [14]. Future investigations on the role of subolesin in *O. turicata* gene expression would reveal important insights on several aspects of blood feeding and reproduction in these ticks.

The analysis of *O. turicata* subolesin amino acid sequence at SignalP 4.1 and T MHMM v.2.0 servers revealed neither the presence of Signal peptide nor the Transmembrane helices respectively, suggesting intracellular localization of this protein. Analysis of *O. turicata* subolesin sequence at TargetP server did not reveal its localization to the membrane or mitochondria. However, prediction of *O. turicata* subolesin sequence using NucPred server [28] showed strong nuclear localization signal, suggesting its role in gene transcription (Additional file 1: Figure S3). The suggested role of subolesin from hard ticks in gene transcription strongly supports its nuclear localization [14]. Based on the sequence identity with other hard ticks, we hypothesize that *O. turicata* subolesin might also localize to the nucleus and function in tick gene transcription. Myristoylation of proteins results in protein-protein interactions and protein-lipid interactions that play an important role in membrane targeting [29]. The absence of myristoylation site (based on the prediction) in *O. turicata* subolesin further supports its intracellular/cytosolic localization. The high percentage identity of various arthropods subolesin orthologs with vertebrate proteins raises a question for its safe efficacy in vaccination trials [30]. However, several studies have immunized vertebrates with intracellular proteins that suggested low risk of autoimmune responses [10,30-33]. Taken together, our study provides additional insights in to the consideration of subolesin as a single vaccine candidate for targeting various vectors that transmit human pathogens.

Phosphorylation is an important post-translational modification that acts as a molecular switch to turn “On” and “Off” controlling activity and function of several proteins involved in cell signaling and/or transcription [24,34,35]. Even though there is no experimental evidence that prove phosphorylation of tick subolesins, human ortholog of tick subolesin has shown to be phosphorylated [36]. The prediction of both PKC and CKII phosphorylation sites suggests that *O. turicata* subolesin might get phosphorylated *in vivo* to perform its function in the regulation of genes important for blood feeding and digestion. The observation of peak levels of subolesin transcripts in post-fed *O. turicata* ticks supports this hypothesis (Figure 6C). Future studies would unravel whether phosphorylation of *O. turicata* subolesin is an important post-translational modification required for its role in gene transcription that may subsequently influence blood feeding, blood digestion and reproduction in these ticks.

## Conclusions

The development of a single vaccine targeting several tick species throughout the world to prevent transmission of pathogens to humans and animals is challenging. However, the current study along with other published studies indicate that subolesin is an important evolutionarily conserved molecule in various tick species and suggests its role in the regulation of genes involved in arthropod immune responses, blood feeding and digestion and reproduction. Disrupting function of this important molecule by vaccines offers an efficient means for controlling several tick vectors before they can transmit harmful disease causing microbes to humans and animals.

## Additional file

Additional file 1: Figure S1. Alignment of *O. turicata* subolesin nucleotide (A) and amino acid sequences (B) of three sequenced clones. **Figure S2.** Serine-rich region prediction in subolesin sequences. **Figure S3.** Nuclear localization signal prediction of *O. turicata* subolesin.

## Abbreviations

PCR: Polymerase chain reaction
QRT-PCR: Quantitative real-time polymerase chain reaction
CK2: Casein kinase II
PKC: Protein Kinase C
LB medium: Luria-Bertani medium

## References

[1] Anderson JF, Magnarelli LA (2008). Biology of ticks. Infect Dis Clin North Am.

[2] Horak IG, Camicas JL, Keirnas JE (2002). The Argasidae, Ixodidae and Nuttalliellidae (Acari: Ixodida): a world list of valid tick names. Exp Appl Acarol.

[3] Sonenshine DE, Roe R. Biology of Ticks, Second Edition. Oxford University Press 2014;1. p. 3–16.

[4] Sonenshine DE, Goodman DTDJL, Sonenshine DE (2005). The Biology of Tick Vectors of Human Disease. Tick-Borne Diseases of Human.

[5] George JE, Pound JM, Davey RB, Bowman AS, Nuttall P (2008). Acaricides for controlling ticks on cattle and the problem of acaricide resistance. Ticks: Biology.

[6] de la Fuente J, Garcia-Garcia JC, Gonzalez DM, Izquierdo G, Ochagavia ME (2000). Molecular analysis of Boophilus spp. (Acari: Ixodidae) tick strains. Vet Parasitol.

[7] Sossai S, Peconick AP, Sales-Junior PA, Marcelino FC, Vargas MI, Neves ES (2005). Polymorphism of the bm86 gene in South American strains of the cattle tick Boophilus microplus. Exp Appl Acarol.

[8] Garcia-Garcia JC, Gonzalez IL, Gonzalez DM, Valdes M, Mendez L, Lamberti J (1999). Sequence variations in the Boophilus microplus Bm86 locus and implications for immunoprotection in cattle vaccinated with this antigen. Exp Appl Acarol.

[9] Popara M, Villar M, Mateos-Hernandez L, de Mera IG, Marina A, del Valle M (2013). Lesser protein degradation machinery correlates with higher BM86 tick vaccine efficacy in Rhipicephalus annulatus when compared to Rhipicephalus microplus. Vaccine.

[10] Almazan C, Kocan KM, Bergman DK, Garcia-Garcia JC, Blouin EF, de la Fuente J (2003). Identification of protective antigens for the control of Ixodes scapularis infestations using cDNA expression library immunization. Vaccine.

[11] de la Fuente J, Almazan C, Blouin EF, Naranjo V, Kocan KM (2005). RNA interference screening in ticks for identification of protective antigens. Parasitol Res.

[12] de la Fuente J, Almazan C, Blas-Machado U, Naranjo V, Mangold AJ, Blouin EF (2006). The tick protective antigen, 4D8, is a conserved protein involved in modulation of tick blood ingestion and reproduction. Vaccine.

[13] Manzano-Roman R, Diaz-Martin V, Oleaga A, Siles-Lucas M, Perez-Sanchez R (2012). Subolesin/akirin orthologs from Ornithodoros spp. soft ticks: cloning, RNAi gene silencing and protective effect of the recombinant proteins. Vet Parsitol.

[14] de la Fuente J, Maritz-Olivier C, Naranjo V, Ayoubi P, Nijhof AM, Almazan C (2008). Evidence of the role of tick subolesin in gene expression. BMC Genomics.

[15] de la Fuente J, Moreno-Cid JA, Galindo RC, Almazan C, Kocan KM, Merino O (2013). Subolesin/Akirin vaccines for the control of arthropod vectors and vector-borne pathogens. Transbound Emerg Dis.

[16] Harrington D, Canales M, de la Fuente J, de Luna C, Robinson K, Guy J (2009). Immunisation with recombinant proteins subolesin and Bm86 for the control of Dermanyssus gallinae in poultry. Vaccine.

[17] Moreno-Cid JA, Jimenez M, Cornelie S, Molina R, Alarcon P, Lacroix MN (2010). Characterization of Aedes albopictus akirin for the control of mosquito and sand fly infestations. Vaccine.

[18] Carreon D, de la Lastra JM, Almazan C, Canales M, Ruiz-Fons F, Boadella M (2012). Vaccination with BM86, subolesin and akirin protective antigens for the control of tick infestations in white tailed deer and red deer. Vaccine.

[19] Davis GE (1941). Ornithodoros turicata:the males; feeding and copulation habits, fertility, span of life, and the transmission of relapsing fever spirochetes. Pub Health Rep.

[20] Cutler SJ, Abdissa A, Trape JF (2009). New concepts for the old challenge of African relapsing fever borreliosis. Clin Microbiol Infect: the official publication of the European Society of Clinical Microbiology and Infectious Diseases.

[21] Estrada-Pena A, Jongejan F (1999). Ticks feeding on humans: a review of records on human-biting Ixodoidea with special reference to pathogen transmission. Exp Appl Acarol.

[22] Burgdorfer W (1956). The possible role of ticks as vectors of leptospirae. I. Transmission of Leptospira pomona by the argasid tick, Ornithodoros turicata, and the persistance of this organism in its tissues. Exp Parasitol.

[23] Neelakanta G, Sultana H, Fish D, Anderson JF, Fikrig E (2010). Anaplasma phagocytophilum induces Ixodes scapularis ticks to express an antifreeze glycoprotein gene that enhances their survival in the cold. J Clin Invest.

[24] Sultana H, Neelakanta G, Kantor FS, Malawista SE, Fish D, Montgomery RR (2010). Anaplasma phagocytophilum induces actin phosphorylation to selectively regulate gene transcription in Ixodes scapularis ticks. J Exp Med.

[25] Odongo D, Kamau L, Skilton R, Mwaura S, Nitsch C, Musoke A (2007). Vaccination of cattle with TickGARD induces cross-reactive antibodies binding to conserved linear peptides of Bm86 homologues in Boophilus decoloratus. Vaccine.

[26] Valle MR, Mendez L, Valdez M, Redondo M, Espinosa CM, Vargas M (2004). Integrated control of Boophilus microplus ticks in Cuba based on vaccination with the anti-tick vaccine Gavac. Exp Appl Acarol.

[27] Willadsen P, Smith D, Cobon G, McKenna RV (1996). Comparative vaccination of cattle against Boophilus microplus with recombinant antigen Bm86 alone or in combination with recombinant Bm91. Parsit Immunol.

[28] Brameier M, Krings A, MacCallum RM (2007). NucPred–predicting nuclear localization of proteins. Bioinformatics.

[29] Farazi TA, Waksman G, Gordon JI (2001). The biology and enzymology of protein N-myristoylation. J Biol Chem.

[30] Almazan C, Blas-Machado U, Kocan KM, Yoshioka JH, Blouin EF, Mangold AJ (2005). Characterization of three Ixodes scapularis cDNAs protective against tick infestations. Vaccine.

[31] Elad D, Segal E (1995). Immunogenicity in calves of a crude ribosomal fraction of Trichophyton verrucosum: a field trial. Vaccine.

[32] Almazan C, Lagunes R, Villar M, Canales M, Rosario-Cruz R, Jongejan F (2010). Identification and characterization of Rhipicephalus (Boophilus) microplus candidate protective antigens for the control of cattle tick infestations. Parasitol Res.

[33] Canales M, Naranjo V, Almazan C, Molina R, Tsuruta SA, Szabo MP (2009). Conservation and immunogenicity of the mosquito ortholog of the tick-protective antigen, subolesin. Parsitol Res.

[34] Mahesh G, Jeong E, Ng FS, Liu Y, Gunawardhana K, Houl JH (2014). Phosphorylation of the transcription activator CLOCK regulates progression through a approximately 24-h feedback loop to influence the circadian period in Drosophila. J Biol Chem.

[35] Comer AR, Ahern-Djamali SM, Juang JL, Jackson PD, Hoffmann FM (1998). Phosphorylation of Enabled by the Drosophila Abelson tyrosine kinase regulates the *in vivo* function and protein-protein interactions of Enabled. Mol Cell Biol.

[36] Olsen JV, Blagoev B, Gnad F, Macek B, Kumar C, Mortensen P (2006). Global, *in vivo*, and site-specific phosphorylation dynamics in signaling networks. Cell.

