# Supplementary material for: Identification and comparative analysis of subolesin/akirin ortholog from Ornithodoros turicata ticks
Source: Parasit Vectors. 2015 Feb 28;8:132. doi: 10.1186/s13071-015-0749-x (PMC4359563; doi:10.1186/s13071-015-0749-x)
Supplement: Additional file 1: Figure S1. — Alignment of O. turicata subolesin nucleotide (A) and amino acid sequences (B) of three sequenced clones. Figure S2. Serine-rich region prediction in subolesin sequences. Figure S3. Nuclear localization signal prediction of O. turicata subolesin. [file 13071_2015_749_MOESM1_ESM.pdf]

Supplementary Figure legends

Supplementary Figure 1. Alignment of *O. turicata* subolesin nucleotide (A) and amino acid sequences (B) of three sequenced clones. The Clustal W alignment of nucleotide (A) and amino-acid sequences (B) is shown. Residues that match are shaded with black color. Consensus sequences for alignments are shown on the top of the rulers.

Supplementary Figure 2. Serine-rich region prediction in subolesin sequences. Annotated amino acid sequences of several subolesins were individually analyzed at PROSITE database for serine-rich region sites. Histograms represent number of serine-rich region sites for each subolesin. Organism names and groups are shown at the bottom of the figure.

Supplementary Figure 3. Nuclear localization signal prediction of *O. turicata* subolesin. *O. turicata* annotated amino acid sequence was analyzed at NucPred server for nuclear localization signal prediction. The analysis revealed a score of 0.93 indicating a strong nuclear signal in *O. turicata* subolesin sequence. Color coding scale was obtained from NucPred analysis.

A

```
Majority TGGCTTGTGCAACATTAAAGCGAACGCATGATTGGGATCCTTTGCACAGTCCCAATGGACGGGCACCTAAGCGAAGGCGGTGTATGCCACTGTGCGTTTCACCGTCAACTCCACCATCTCGGGCGCACCAGATCTGTCCGTCACCTTTCG
                  10        20        30        40        50        60        70        80        90       100       110       120       130       140       150
Clone-1  TGGCTTGTGCAACATTAAAGCGAACGCATGATTGGGATCCTTTGCACAGTCCCAATGGACGGGCACCTAAGCGAAGGCGGTGTATGCCACTGTGCGTTTCACCGTCAACTCCACCATCTCGGGCGCACCAGATCTGTCCGTCACCTTTCG 150
Clone-2  TGGCTTGTGCGACATTAAAGCGAACGCATGATTGGGATCCTTTGCACAGTCCCAATGGACGGGCACCTAAGCGAAGGCGGTGTATGCCACTGTGCGTTTCACCGTCAACTCCACCATCTCGGGCGCACCAGATCTGTCCGTCACCTTTCG 150
Clone-3  TGGCTTGTGCAACATTAAAGCGAACGCATGATTGGGATCCTTTGCACAGTCCCAATGGACGGGCACCTAAGCGAAGGCGGTGTATGCCACTGTGCGTTTCACCGTCAACTCCACCATCTCGGGCGCACCAGATCTGTCCGTCACCTTTCG 150

Majority TTGAAGTGCCACCGAAGCTCTCTTCAGAGGAAATTGCTGCCAACATTCGGGAAGAAATGAAACGGTTGCAGCGGCGCAAGCAACTGTGCTTTCCGACGCTTGATTCCTCTCCACAGAGTGCAGACTCCCTTCCCAGCTCACCCACAGGAG
                 160       170       180       190       200       210       220       230       240       250       260       270       280       290       300
Clone-1  TTGAAGTGCCACCGAAGCTCTCTTCAGAGGAAATTGCTGCCAACATTCGGGAAGAAATGAAACGGTTGCAGCGGCGCAAGCAACTGTGCTTTCCGACGCTTGATTCCTCTCCACAGAGTGCAGACTCCCTTCCCAGCTCACCCACAGGAG 300
Clone-2  TTGAAGTGCCACCGAAGCTCTCTTCAGAGGAAATTGCTGCCAACATTCGGGAAGAAATGAAACGGTTGCAGCGGCGCAAGCAACTGTGCTTTCCGACGCTTGATTCCTCTCCACAGAGTGCAGACTCCCTTCCCAGCTCACCCACAGGAG 300
Clone-3  TTGAAGTGCCACCGAAGCTCTCTTCAGAGGAAATTGCTGCCAACATTCGGGAAGAAATGAAACGGTTGCAGCGGCGCAAGCAACTGTGCTTTCCGACGCTTGATTCCTCTCCACAGAGTGCAGACTCCCTTCCCAGCTCACCCACAGGAG 300

Majority GACTTCTGTCTCCTGTGCGAAGGGACCAACCTCTGTTCACCTTTCGTCAAGTAGGACTCATCTGTGAACGAATGATGAAAGAGCGTGAATGCCAGATCAGGGAACAGTATGACCAAGTTCTCTCTACCAAGCTTGCAGAGCAGTACGACA
                 310       320       330       340       350       360       370       380       390       400       410       420       430       440       450
Clone-1  GACTTCTGTCTCCTGTGCGAAGGGACCAACCTCTGTTCACCTTTCGTCAAGTAGGACTCATCTGTGAACGAATGATGAAAGAGCGTGAATGCCAGATCAGGGAACAGTATGACCAAGTTCTCTCTACCAAGCTTGCAGAGCAGTACGACA 450
Clone-2  GACTTCTGTCTCCTGTGCGAAGGGACCAACCTCTGTTCACCTTTCGTCAAGTAGGACTCATCTGTGAACGAATGATGAAAGAGCGTGAATGCCAGATCAGGGAACAGTATGACCAAGTTCTCTCTACCAAGCTTGCAGAGCAGTACGACA 450
Clone-3  GACTTCTGTCTCCTGTGCGAAGGGACCAACCTCTGTTCACCTTTCGTCAAGTAGGACTCATCTGTGAACGAATGATGAAAGAGCGTGAATGCCAGATCAGGGAACAGTATGACCAAGTTCTCTCTACCAAGCTTGCAGAGCAGTACGACA 450

Majority CATTTGTCAAGTTTACCTACGACCAAA
                 460       470
Clone-1  CATTTGTCAAGTTTACCTACGACCAAA 477
Clone-2  CATTTGTCAAGTTTACCTACGACCAAA 477
Clone-3  CATTTGTCAAGTTTACCTACGACCAAA 477
```

B

```
Majority    ACATLKRTHDWDPLHSPNGRAPKRRRCMPLCVSPSTPPSRAHQICPSPFVEVPPKLSSEEIAANIREEMKRLQRRKQLCFPTLDSSPQSADSLPSSPTGG
                     10        20        30        40        50        60        70        80        90       100
Clone-1.pro ACATLKRTHDWDPLHSPNGRAPKRRRCMPLCVSPSTPPSRAHQICPSPFVEVPPKLSSEEIAANIREEMKRLQRRKQLCFPTLDSSPQSADSLPSSPTGG 100
Clone-2.pro ACATLKRTHDWDPLHSPNGRAPKRRRCMPLCVSPSTPPSRAHQICPSPFVEVPPKLSSEEIAANIREEMKRLQRRKQLCFPTLDSSPQSADSLPSSPTGG 100
Clone-3.pro ACATLKRTHDWDPLHSPNGRAPKRRRCMPLCVSPSTPPSRAHQICPSPFVEVPPKLSSEEIAANIREEMKRLQRRKQLCFPTLDSSPQSADSLPSSPTGG 100

Majority    LLSPVRRDQPLFTFRQVGLICERMMKERECQIREQYDQVLSTKLAEQYDTFVKFTYDQ
                    110       120       130       140       150
Clone-1.pro LLSPVRRDQPLFTFRQVGLICERMMKERECQIREQYDQVLSTKLAEQYDTFVKFTYDQ 158
Clone-2.pro LLSPVRRDQPLFTFRQVGLICERMMKERECQIREQYDQVLSTKLAEQYDTFVKFTYDQ 158
Clone-3.pro LLSPVRRDQPLFTFRQVGLICERMMKERECQIREQYDQVLSTKLAEQYDTFVKFTYDQ 158
```

Supplementary Figure 1

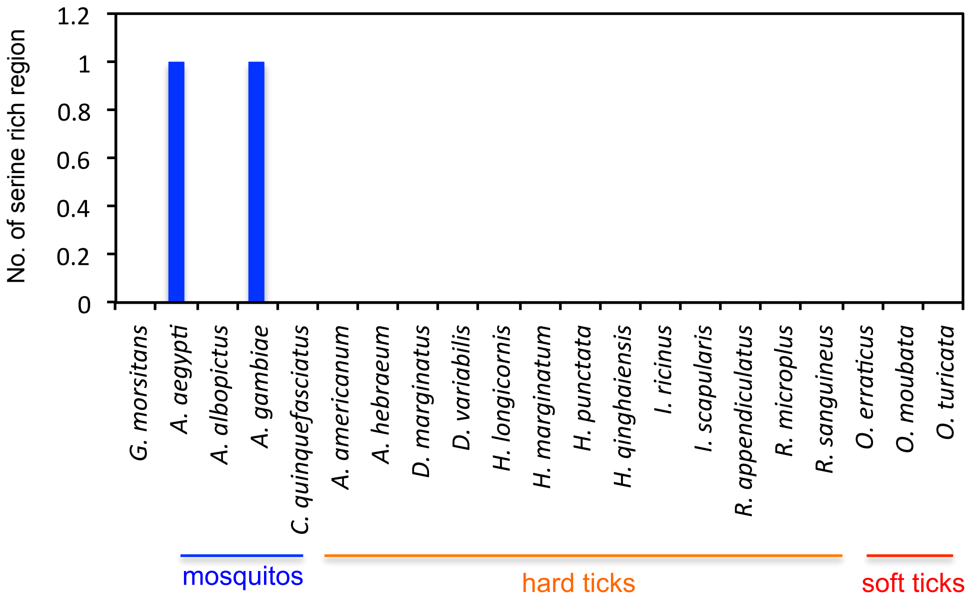

Supplementary Figure 2

1	ACATLKRTHDWDPLHSPNGRAPKRRRCMPLCVSPSTPPSRAHQICPSPFV	50
51	EVPPKLSSEEIAANIREEMKRLQRRKQLCFPTLDSSPQSADSLPSSPTGG	100
101	LLSPVRRDQPLFTFRQVGLICERMMKERECQIREQYDQVLSTKLAEQYDT	150
151	FVKFTYDQ	158

Positively and negatively influencing subsequences are coloured according to the following scale:

(non-
nuclear) negative 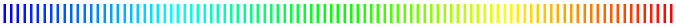 positive (nuclear)

Supplementary Figure 3
